# Rational metareasoning and the plasticity of cognitive control

**DOI:** 10.1371/journal.pcbi.1006043

**Published:** 2018-04-25

**Authors:** Falk Lieder, Amitai Shenhav, Sebastian Musslick, Thomas L. Griffiths

**Affiliations:** 1 Helen Wills Neuroscience Institute, University of California, Berkeley, Berkeley, California, United States of America; 2 Brown Institute for Brain Science, Department of Cognitive, Linguistic, and Psychological Sciences, Brown University, Providence, Rhode Island, United States of America; 3 Princeton Neuroscience Institute, Princeton University, Princeton, New Jersey, United States of America; 4 Institute for Cognitive and Brain Sciences, Department of Psychology, University of California, Berkeley, Berkeley, California, United States of America; Harvard University, UNITED STATES

## Abstract

The human brain has the impressive capacity to adapt how it processes information to high-level goals. While it is known that these cognitive control skills are malleable and can be improved through training, the underlying plasticity mechanisms are not well understood. Here, we develop and evaluate a model of how people learn when to exert cognitive control, which controlled process to use, and how much effort to exert. We derive this model from a general theory according to which the function of cognitive control is to select and configure neural pathways so as to make optimal use of finite time and limited computational resources. The central idea of our Learned Value of Control model is that people use reinforcement learning to predict the value of candidate control signals of different types and intensities based on stimulus features. This model correctly predicts the learning and transfer effects underlying the adaptive control-demanding behavior observed in an experiment on visual attention and four experiments on interference control in Stroop and Flanker paradigms. Moreover, our model explained these findings significantly better than an associative learning model and a Win-Stay Lose-Shift model. Our findings elucidate how learning and experience might shape people’s ability and propensity to adaptively control their minds and behavior. We conclude by predicting under which circumstances these learning mechanisms might lead to self-control failure.

## Author summary

The human brain has the impressive ability to adapt how it processes information to high level goals. While it is known that these cognitive control skills are malleable and can be improved through training, the underlying plasticity mechanisms are not well understood. Here, we derive a computational model of how people learn when to exert cognitive control, which controlled process to use, and how much effort to exert from a formal theory of the function of cognitive control. Across five experiments, we find that our model correctly predicts that people learn to adaptively regulate their attention and decision-making and how these learning effects transfer to novel situations. Our findings elucidate how learning and experience might shape people’s ability and propensity to adaptively control their minds and behavior. We conclude by predicting under which circumstances these learning mechanisms might lead to self-control failure.

## Introduction

The human brain has the impressive ability to adapt how it processes information and responds to stimuli in the service of high level goals, such as writing an article [1]. The mechanisms underlying this behavioral flexibility range from seemingly simple processes, such as inhibiting the impulse to browse your Facebook feed, to very complex processes such as orchestrating your thoughts to reach a solid conclusion. Our capacity for *cognitive control* enables us to override automatic processes when they are inappropriate for the current situation or misaligned with our current goals. One of the paradigms used to study cognitive control is the Stroop task, where participants are instructed to name the hue of a color word (e.g., respond “green” when seeing the stimulus RED) while inhibiting their automatic tendency to read the word (“red”) [2]. Similarly, in the Eriksen flanker task, participants are asked to report the identity of a target stimulus surrounded by multiple distractors while overcoming their automatic tendency to respond instead to the distractors. Individual differences in the capacity for cognitive control are highly predictive of academic achievement, interpersonal success, and many other important life outcomes [3,4].

While exerting cognitive control improves people’s performance in these tasks, it is also effortful and appears to be intrinsically costly [5,6]. The Expected Value of Control (EVC) theory maintains that the brain therefore specifies how much control to exert according to a rational cost-benefit analysis, weighing these effort costs against attendant rewards for achieving one’s goals [7]. In broad accord with the predictions of the EVC theory, previous research has found that control specification is context-sensitive [8,9] and modulated by reward across multiple domains [10,11], such as attention, response inhibition, interference control, and task switching. While previous theories account for that fact that people’s performance in these task is sensitive to reward [7,12–14], it remains unclear *how* these dependencies arise from people’s experience. Recently, it has been proposed that the underlying mechanism is associative learning [15,16]. Indeed, a number of studies have demonstrated that cognitive control specification is plastic: whether people exert cognitive control in a given situation, which controlled processes they employ, and how much control they allocate to them is learned from experience. For instance, it has been demonstrated that participants in visual search tasks gradually learn to allocate their attention to locations whose features predict the appearance of a target [17], and a recent study found that learning continuously adjusts how much cognitive control people exert in a Stroop task with changing difficulty [18]. Furthermore, it has been shown that people learn to exert more cognitive control after their performance on a control-demanding task was rewarded [10] and learn to exert more control in response to potentially control-demanding stimuli that are associated with reward than to those that are not [11].

These studies provide evidence that people can use information from their environment (e.g., stimulus features) to learn when to exert cognitive control and how to exert control, and it has recently been suggested that this can be thought of in terms of associative learning [15,16]. Other studies suggested that cognitive control can be improved through training [19–21]. However, achieving transfer remains challenging [22–25], the underlying learning mechanisms are poorly understood, and there is currently no theory that could be used to determine which training regimens will be most effective and which real-life situations the training will transfer to. Developing precise computational models of the plasticity of cognitive control may be a promising way to address these problems and to enable more effective training programs for remediating executive dysfunctions and enabling people to pursue their goals more effectively.

In this article, we extend the EVC theory to develop a theoretical framework for modeling the function and plasticity of cognitive control specification. This extension incorporates recent theoretical advances inspired by the rational metareasoning framework developed in the artificial intelligence literature [26,27]. We leverage the resulting framework to derive the Learned Value of Control (LVOC) model which can learn to efficiently select control signals based on features of the task environment. The LVOC model can be used to simulate cognitive control (e.g., responding to a goal-relevant target that competes with distractors) and, more importantly, how it is shaped by learning. According to the LVOC model, people learn the value of different cognitive control signals (e.g., how much to attend one stimulus or another). A key strength of this model is that it is very general and can be applied to phenomena ranging from simple learning effects in the Stroop task to the acquisition of complex strategies for reasoning and problem-solving. In order to demonstrate the validity and generality of this model, we show that it can capture the empirical findings of five cognitive control experiments on the plasticity of visual attention [17], the interacting effects of reward and task difficulty on the plasticity of interference control [10,11], and the transfer of such learning to novel stimuli [8,9]. Moreover, the LVOC model outperforms alternate models of such learning processes that rely only on associative learning or a basic win-lose-stay-shift strategy. Our findings shed light on how learning and experience might shape people’s ability and propensity to adaptively control their minds and behavior, and the LVOC model predicts under which circumstances these mechanisms might lead to self-control failure.

## Models

### Formalizing the function of cognitive control

At an abstract level, all cognitive control processes serve the same function: to adapt neural information processing to achieve a goal [28]. At this abstract level, neural information processing can be characterized by the computations being performed, and the extent to which the brain achieves its goals can be quantified by the expected utility of the resulting actions. From this perspective, an important function of cognitive control is to select computations so as to maximize the agent’s reward rate (i.e., reward per unit time). This problem is formally equivalent to the *rational metareasoning* [26,29] problem studied in computer science: selecting computations so as to make optimal use of the controlled system’s limited computational resources (i.e., to achieve the highest possible sum of rewards with a limited amount of computation).

Thus, rational metareasoning suggests that the specification of cognitive control is a metacognitive decision problem. In reinforcement learning [30], decision problems are typically defined by a set of possible actions, the set of possible states, an initial state, the conditional probabilities of transitioning from one state to another depending on the action taken by the agent, and a reward function. Together these five components define a Markov decision process (MDP [30]). In a typical application of this framework the agent is an animal, robot, or computer program, actions are behaviors (e.g., pressing a lever), the state characterizes the external environment ℰ (e.g., the rat’s location in the maze), and the rewards are obtained from the environment (e.g., pressing a lever dispenses cheese). In general, the agent cannot observe the state of the environment directly; for instance, the rat running through a maze does not have direct access to its location but has to infer this from sensory observations. The decision problems posed by an environment that is only partially observable can be modelled as a partially observable MDP (POMDP [31]). For each POMDP there is an equivalent MDP whose state encodes what the agent knows about the environment and is thus fully observable; this is known as the belief-MDP [31].

Critically, the belief-MDP formalism can also be applied to the choice of internal computations [27]–such as allocating attention [32] or gating information into working memory [33,34]–rather than only physical actions. In the rational metareasoning framework, the agent is the cognitive control system whose actions are control signals that specify which computations the controlled systems should perform. The internal state of the controlled systems is only partially observable. We can formally define the problem of optimal cognitive control specification as maximizing reward the in the meta-level MDP

$$M=(S,s_{0},C,T,r),$$ (1)

where S is the set of possible information states, comprising beliefs about the external environment (e.g., the choices afforded by the current situation) and beliefs about the agent’s internal state (e.g., the decision system’s estimates of the choices’ utilities), *s*_0_ denotes the initial information state, C is the set of possible control signals that may be discrete (e.g., “Simulate action 1.”) or continuous (e.g., “Increase the decision threshold by 0.175.” or “Suppress the activity of the word-reading pathway by 75%.”), *T* is a transition model, and *r* is the reward function that cognitive control seeks to maximize. The transition model specifies the conditional probability of transitioning from belief state *s* to belief state *s*′ if the control signal is *c* by *T*(*s*, *c*, *s*′). The meta-level reward function *r* combines the utility of outcome *X* (of actions resulting from control signal *c* in belief state *s*) with the computational cost associated with exerting cognitive control:

r(s,c)=u(X)-cost(s,c), (2)

where *X* is the outcome of the resulting action, *u* is utility function of the brain’s reward system, and cost(*s*, *c*) is the cost of implementing the controlled process.

Within this framework, we can define a cognitive control strategy π:S→C as a mapping from belief states s∈S to control signals c∈C. The optimal cognitive control strategy *π*^⋆^ is the one that always chooses the computation with the highest expected value of computation (EVOC):

$$\pi^{⋆}:s\mapsto argmax_{c}\;EVOC(c,s).$$ (3)

The EVOC is the expected sum of computational costs and benefits of performing the computation specified by the control signal *c* and continuing optimally from there on:

$$EVOC(c,s)=Q_{\pi^{⋆}}(s,c)=E[r(s,c)+V_{\pi^{⋆}}(S_{t+1})|S_{t}=s,C_{k}=c,T],$$ (4)

where $Q_{\pi^{⋆}}$ is known as the Q-function of the optimal control strategy *π*^⋆^, and $V_{\pi^{⋆}}(S_{t+1})$ is the expected sum of meta-level rewards of starting *π*^⋆^ in state *S*_*t*+1_.

In summary, cognitive control specification selects the sequence of cognitive control signals that maximizes the expected sum of rewards of the resulting actions minus the cost of the controlled process. The optimal solution to this problem is given by the optimal control policy *π*^⋆^.

So far, we have assumed that the cognitive control system chooses one control signal at a time, but ***c*** could also be a vector comprising multiple control signals (e.g., one that increases the rate at which evidence is accumulated towards the correct decision via an attentional mechanism and a second one that adjusts the decision threshold). Furthermore, overriding a habit by a well-reasoned decision also requires executing a coordinated sequence of cognitive operations for planning and reasoning. Instead of specifying each of these operations by a separate control signal, the cognitive control system might sometimes use a single control signal to instruct the decision system to execute an entire planning strategy. The rational metareasoning framework allows us to model cognitive strategies as options [35–38]. An option is a policy combined with an initiation set and a termination condition [38]. Options can be treated as if they were elementary computations and elementary computations can be interpreted as options that terminate after the first step. With this extension, the optimal solution to the cognitive control specification problem becomes

$$\pi^{⋆}(s)=\text{arg}\underset{o\in O}{\text{max}}Q^{⋆}(s,o),$$ (5)

where the set of options O may include control strategies and elementary control signals.

Critically, this rational metareasoning perspective on cognitive control covers not only simple phenomena, such as inhibiting a pre-potent automatic response in the Stroop task, but also more complex ones, such as sequencing one’s thoughts so as to follow a good decision strategy, and very complex phenomena such as reasoning about how to best solve a complex problem.

### The LVOC model of the plasticity of cognitive control specification

The computations required to determine the expected value of control may themselves be costly and time consuming. Yet, in some situations cognitive control has to be engaged very rapidly, because maladaptive reflexes, impulses, and habitual responses have to be inhibited before the triggered response has been executed. In such situations, there is simply not enough time to compute the expected value of control on the fly. Fortunately, this may not be necessary because an approximation to the EVOC can be learned from experience. We therefore hypothesize that the cognitive control system learns to predict the context-dependent value of alternative control signals. By understanding how this learning occurs, we might be able to explain the experience-dependent changes in how people use their capacity for cognitive control, which we will refer to as the plasticity of cognitive control specification. In addition to these systematic, experience-driven changes cognitive control is also intrinsically variable. To model the plasticity and the variability of cognitive control, this section develops a model that combines a novel feature-based learning mechanism with a new control specification mechanism that explores promising control signals probabilistically to accelerate learning which of them is most effective.

The previous section characterized the problem of cognitive control specification as a sequential meta-decision problem. This makes reinforcement learning algorithms [39] a natural starting point for exploring how the cognitive control systems learns the EVOC from experience. Approximate Q-learning appears particularly suitable because the optimal control strategy can be expressed in terms of the optimal Q-function (Eqs 3–5). From this perspective, the plasticity mechanisms of cognitive control specification serve to learn an approximation to the value *Q*_*t*_(*s*, *c*) of selecting control signal *c* in state *s* based on one’s experience with selecting control signals ***c*** = (*c*_1_,⋯,*c*_*t*_) in states ***s*** = (*s*_1_,⋯,*s*_*t*_) and receiving the meta-level rewards ***r*** = (*r*_1_,⋯,*r*_*t*_). Learning an approximate Q-function *Q*_*t*_ from this information could enable the cognitive control system to efficiently select a control strategy by comparing learned values rather than reasoning about their effects.

Learning the optimal meta-level state-value function *Q*^⋆^ can be challenging because the value of each control signal may depend on the outcomes of the control signals selected afterwards. Furthermore, the state space of the meta-level MDP has a very high dimensionality as it comprises all possible states that the controlled system could be in. To overcome these challenges, a neural system like the brain might learn a linear approximation to the meta-level state value function instead of estimating each of its entries separately. Concretely, the cognitive control system might learn to predict the value of selecting a control strategy (e.g., focusing on the presenting speaker instead of attending to an incoming phone call) by a weighted sum of features of the internal state and the current context (e.g. being in a conference room). For instance, the value Q^⋆^(*s*, *c*) of choosing control signal *c* in the internal state *s* can be predicted from the features *f*_*k*_(*s*), the implied control signal intensities ***c***, their interactions with the features, that is *f*_*k*_(*s*) *c*_*i*_, and their costs. Concretely, the EVOC of selecting control signal *c* in state *s* is approximated by the *Learned Value of Control* (LVOC),

$$\begin{array}{ll} \text{LVOC}\left(s,c;w\right) & =w_{0}+\left(\sum_{k=1}^{K}w_{k}^{\left(f\right)}\cdot f_{k}\left(s\right)\right)+\left(\sum_{l=1}^{L}w_{l}^{\left(c\right)}\cdot c_{l}\right)\\ & +\left(\sum_{k=1}^{K}\sum_{l=1}^{L}w_{k,l}^{\left(f\times c\right)}\cdot f_{k}\left(s\right)\cdot c_{l}\right)-\text{cost}\left(c\right)-w^{\left(T\right)}\cdot T, \end{array}$$ (6)

where the weight vector ***w*** includes the offset *w*_0_, the weights $w_{k}^{\left(f\right)}$ of the states’ features, the weights ***w***^(*c*)^ of the control signal intensities, the weights $w_{k,l}^{\left(f\times c\right)}$ of their interaction terms, the weight *w*^(*T*)^ of the response time *T*, and cost(*c*) is the intrinsic cost of control which scales with the amount of cognitive control applied to the task.

The optimal way to update the weights based on experience in a stationary environment is given by Bayes rule. Our model therefore maintains and continues to update an approximation to the posterior distribution

$$P(w|e_{1,\cdots ,t})\propto P(w|e_{1,\cdots ,t-1})\cdot P(e_{t}|w),$$ (7)

on the weight vector ***w*** given its experience *e*_1,⋯,*t*_ up until the present time *t*, where each experience *e*_*i*_ = (*s*_*i*_, *c*_*i*_, *r*_*i*_, *T*_*i*_, *s*_*i*+1_) comprises the state, the selected control signal, the reward, the response time, and the next state. In simple settings where a single control signal determines a single reward our model’s learning mechanism is equivalent to Bayesian linear regression [40,41]. In more complex settings involving a series of control signals or delayed rewards the learning rule approximates the Bayesian update by substituting the delayed costs and benefits of control by the model’s predictions. For more details, see S1 Text.

If the value of control is initially unknown, the optimal way to select control signals is to balance exploiting previous experience to maximize the expected immediate performance with exploring alternative control allocations that might prove even more effective. Our model solves this dilemma by an exploration strategy similar to Thompson sampling: It draws *k* samples from the posterior distribution on the weights and averages them, that is

$${\tilde{w}}_{1},\cdots ,{\tilde{w}}_{k}∼P(w|e_{1,\cdots ,t}),\;\;\;\;\tilde{w}=\frac{1}{k}\cdot \sum_{i=1}^{k}{\tilde{w}}_{i}.$$ (8)

According to the LVOC model the brain then selects a control signal by maximizing the EVOC predicted by the average weight $\tilde{w}$, that is

$$c_{t}\approx {\text{arg}max}_{c}\;LVOC(s_{t},c;\tilde{\;w}).$$ (9)

Together, Eqs 6–9 define the LVOC model of the plasticity of cognitive control. The LVOC model extends the EVC theory [7] which defines optimal control signals in terms of the EVOC (Eq 3), by proposing two mechanisms through which the brain might be able to approximate this normative ideal: learning a feature-based, probabilistic model of the EVOC (Eqs 6 and 7) and selecting control signals by sampling from this model (Eqs 8 and 9). This model is very general and can be applied to model cognitive control of many different processes (e.g., which location to saccade to vs. how strongly to inhibit the word-reading pathway) and different components of the same process (e.g., rate of evidence accumulation towards the correct decision vs. the decision threshold). The LVOC model’s core assumptions are that the brain learns to predict the EVOC of alternative control specifications from features of the situation and the control signals, and that the brain then probabilistically selects the control specification with the highest predicted value of control. Both of these components could be implemented by many different mechanisms. For instance, instead of implementing the proposed approximation to Bayesian regression, the brain might learn to predict the EVOC through the reward-modulated associative plasticity mechanism outlined in the SI. We are therefore not committed to the specific instantiation we used (Eqs 7–9) for the purpose of the simulations reported below.

The LVOC model instantiates the very general theory that the brain learns how to process information via metacognitive reinforcement learning. This includes not only the plasticity of cognitive control but also how people might discover cognitive strategies for reasoning and decision-making and how they learn to regulate their mental activities during problem solving. As a proof of concept, the following sections validate the LVOC model against five experiments on the plasticity of attention and interference control.

### Alternative models: Associative learning and Win-Stay Lose-Shift

In principle, the control-demanding behavior considered in this paper could result from simpler mechanisms than the ones proposed here. In this section, we consider two simple models that we use as alternatives to compare against the more complex LVOC model. The first model relies on the assumption that the plasticity of cognitive control can be understood in terms of associative learning [15,16]. We therefore evaluate our model against an associative learning model based on the Rescorla-Wagner learning rule [42]. This model forms stimulus-control associations based on the resulting reward. The association *A*_*s*,*c*_ between a stimulus *s* and a control signal *c* is strengthened when it is accompanied by (intrinsic or extrinsic) reward and weakened otherwise. Concretely, the association strengths involving the chosen response were updated according to the Rescorla-Wagner rule, that is

$$A_{s,c}=A_{s,c}+\alpha \cdot \left(R-\sum_{s}I_{s}\cdot A_{s,c}\right),$$ (10)

where *α* is the learning rate, *R* is the reward and the indicator variable *I*_*s*_ is 1 when the stimulus *s* was present and 0 else. Given the learned associations, the control signal is chosen probabilistically according to the exponentiated Luce’s choice rule, that is each control signal *c* is selected with probability

$$p(c)=\frac{{exp(A}_{s,c})}{\sum_{c}{exp(A}_{s,c})}.$$ (11)

The second alternative model is based on previous research suggesting that people sequentially adjust their strategy through a simple Win-Stay Lose-Shift mechanism [43]. On the first trial, this mechanism chooses a strategy at random, and on each subsequent trial it either repeats the previous strategy when it was successful or switches to a different strategy when the current strategy failed. Here, we apply this idea to model how the brain learns which control signal to select. Concretely, our WSLS model repeats the previous control signal (e.g., “Attend to green.”) when it leads to a positive outcome (Win-Stay) and randomly selects a different control signal (e.g., “Attend to red.”) otherwise (Lose-Shift).

In contrast to the LVOC mode, the two alternative models assume that control signals are discrete rather than continuous. In the context of visual attention, they choose their control signal *c* from the set {1,2,3,⋯,12} of possible locations to attend, and in the context of inhibitory control they decide to either inhibit the process completely or not at all (*c* ∈ {0,1}).

### Simulations of learning and transfer effects in cognitive control paradigms

To evaluate the proposed models, we used them to simulate the plasticity of attentional control in a visual search task [17] as well as learning and transfer effects in Stroop and Flanker paradigms [8–11]. Table 1 summarizes the simulated phenomena and how the LVOC model explains each at a conceptual level.

**Table 1:** The core assumption of the LVOC model explains the learning effects observed in five different cognitive control experiments.

	Phenomenon	Explanation of the LVOC model
Lin et al. (2016), Exp. 1	In the training block, participants learn to find the target increasingly faster when it always appears in a location with a certain color. In the test block, participants are significantly slower on trials that violate this regularity.	People learn to predict the value of attending to different locations from their color.
Krebs et al. (2010), Exp. 1	People come to name the color of incongruent words faster and more accurately for colors for which performance is rewarded.	People learn to predict the value of increasing control intensity from the color of the word.
Braem et al. (2012), Exp. 1	On a congruent Flanker trial, people are faster when the previous trial was rewarded and congruent than when it was unrewarded and congruent, but the opposite holds when the previous trial was incongruent. These effects are amplified in people with high reward sensitivity.	People learn to exert more control on incongruent trials. Thus, rewarded incongruent trials tend to reinforce higher control signals while rewarded congruent trials tend to reinforce low control signals. Thus, people increase control after the former and lower control after the latter.
Bugg et al. (2008), Exp. 2	People become faster and more accurate at naming the color of an incongruently colored word when it is usually incongruent than when it is usually congruent.	People learn that exerting more control is more valuable when the color or word is predictive of incongruence.
Bugg et al. (2011), Exp. 2	People are faster at naming animals in novel, incongruently labelled images when that species was mostly incongruently labelled in the training phase than when it was mostly congruently labelled.	People learn that exerting more control is more valuable when the semantic category of the picture is predictive of incongruence.

#### Learning to control visual attention

**Fig 1:**
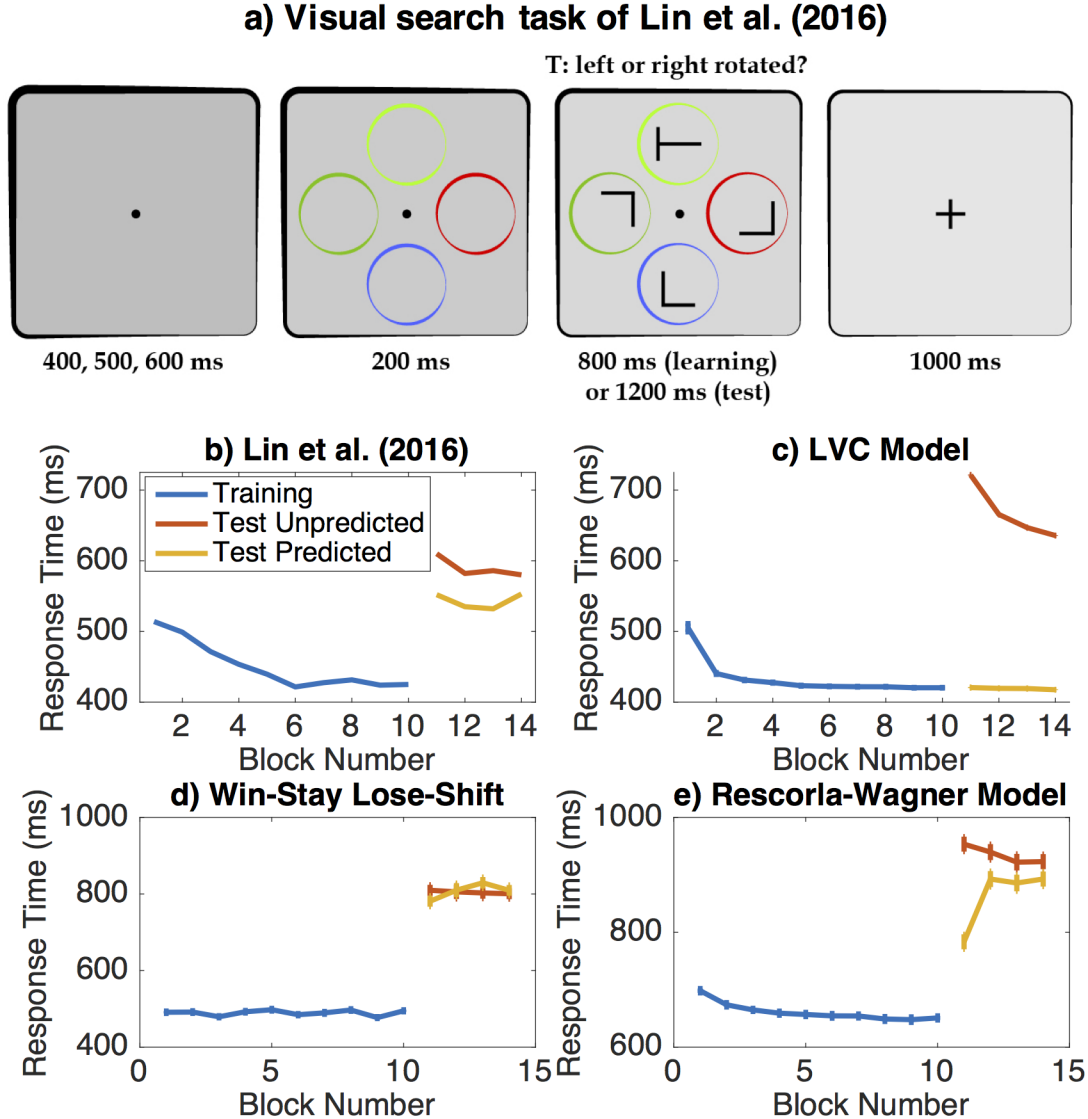
Learning to control the allocation of attention. a) Visual search task used by Lin et al. (2016). b) Human data from Experiment 1 of Lin et al. (2016). c) Predictions of the LVOC model. d) Fit of Win-Stay Lose-Shift model. e) Fit of Rescorla-Wagner model.

Previous research has shown that how people allocate their attention is shaped by learning [8–11,15–17]. For instance, Lin and colleagues [17] had participants perform a visual search task in which they gradually learned to allocate their attention to locations whose color predicted the appearance of the target (Fig 1a). In this task, participants viewed an array of four rotated letters (one T and three L’s), each encompassed by a different colored circle. They were instructed to report the orientation of the T. The circles appeared before the letters allowing participants to allocate their attention by saccading to a promising location before the letters appeared. In the training phase, the target always appeared within the green circle, but in the test phase it was equally likely to appear in any of the four circles.

Visual search entails sequentially allocating cognitive control to different locations based on their visual features. Since attention can be understood as an instance of cognitive control, this problem is naturally modeled as a meta-level MDP. We therefore applied our LVOC theory to predict the dynamics and consequences of learning which locations to attend to based on their features (the colored circles) in this paradigm. Since the stimuli were presented along a circle, approximating locations people might naturally attend around a clock, we assumed that the control signal *c* ∈ {1,2,3,…,12} specifies which of 12 locations to attend, the state *s*_*t*_ encodes which of the 12 locations were highlighted by a colored circle (see Fig 1a), the circles’ colors, the unknown position of the target, and the list of locations that have already been inspected on the current trial. Since the set of possible control signals is small, our simulation assumes that the brain always finds the control signal that maximizes the predicted EVOC (Eq 9).

The features ***f***(*s*, *c*) encode only observable aspects of the state *s* that are relevant to the value of the control signal *c*. Concretely, our simulations assumed that the features encode whether the attended location was highlighted by a colored circle, the color of that circle (one binary indicator variable for each possible color), its position (by one binary indicator variable for each of the four possible locations), and whether or not it has been attended before. To capture people’s prior knowledge that attending a location a second time is unlikely to provide new information, we set the prior on the weight of the last feature to −1; this captures the well-known inhibition of return mechanism in visual attention [44]. For all other features the mean of the prior on the weights was 0. Based on the results reported by [17], we modeled reaction times as the sum of a non-decision time of 319ms and a decision-time of 98ms per attended location. Our simulation assumed that people incur a fixed cost (*r*(*s*, *c*) = cost(c) = −1 for all *c* ∈ {1,2,3,4}) every time they deploy their attention to a location. For simplicity, we assume that in this simple task people always search until they find the target and that when they attend to a location they always recognize the presence/absence of the target and respond accordingly. Hence, the intuition that people should try to find the target with as few saccades as possible follows directly from the objective of maximizing the sum of meta-level rewards. Applied to this visual search task, the LVOC model offers a mechanism for how people learn where to allocate their attention based on environmental cues in order to find the target as quickly as possible.

Our associative learning model assumed that finding the target yields an intrinsic reward of +1 and no reward or cost otherwise. The responses *C* were saccades to one of the 12 locations. The stimuli S comprised indicator variables for each of the four colors, the absence of a circle, and whether the location had been inspected before, and one feature that was always 1. To capture the inhibition of return, the reward associations with the stimulus-feature indicating that a location had been attended previously were initialized to −1. All other association strengths were initialized to 0 and the learning rate of the Rescorla-Wagner model was fit to the data from [17] using maximum-likelihood estimation.

In this task, the WSLS model also produces a sequence of saccades *c*_1_, *c*_2_, ⋯ ∈ *C* by repeatedly shifting its attention to a different location until the target is found.

#### Learning and transfer effects in inhibitory control

In Stroop and Flanker paradigms, the cognitive control strategy *o* is defined by a single control signal *c* ∈ [0,1] which serves to bias processing away from an automatic mechanism. Following the classic model by Cohen and colleagues [45], we assume that control signals determine the relative contribution of the automatic versus the controlled process to the drift rate *d* with which evidence is accumulated towards the controlled response [46]:

$$d=c\cdot d_{controlled}+(1-c)\cdot C\cdot d_{automatic},$$ (12)

where *d*_controlled_ and *d*_automatic_ are the drift rates of the controlled and the automatic process respectively, and *C* = 1 when the trial is congruent or −1 when the trial is incongruent. The drift rates, in turn, affect the response and response time according to a drift-diffusion model [46]. When the decision variable exceeds the threshold +*θ*, then the response agrees with the controlled process (equivalent to the correct response for these tasks). When the decision variable falls below −*θ*, the response is incorrect. To capture sources of error outside of the evidence accumulation process (e.g., motor execution errors), our simulation assumes that people accidentally give the opposite of their intended response on a small fraction of trials (*p*_flip_<0.05).

We model the selection of continuous control signals as a gradient ascent on the EVOC predicted by Thompson sampling (Eqs 8 and 9). Concretely, continuous control signals are selecting by repeatedly applying the update rule

$$c\leftarrow c+\eta \cdot \frac{d\;LVOC(s,c;\;\tilde{w})}{dc}$$ (13)

until the change in the Euclidean norm of the control signal intensity vector is less than *δ*_*c*_. This mechanism starts from the control signal deployed on the previous trial and thereby captures the inertia of control specification [47]. Furthermore, it predicts that control intensities are adjusted gradually and continually, thereby allowing control to be exerted while the optimal control signal is still being determined. This feature of our model makes the intuitive prediction that time pressure might reduce the magnitude of control adjustment [cf. 48,49].

We model the cost associated with a continuous control signal *c* as the sum of the control cost required to exert that amount of control (cost(*c*)) and the opportunity cost of executing the controlled process (*ω*⋅*t*), that is

cost(s,c)=ω⋅t+cost(c), (14)

where *ω* is the opportunity cost per unit time, *t* is the duration of the controlled process, and cost(*c*) is the intrinsic cost of exerting the control signal *c*. While the first term captures that goal-directed control processes, such as planning, can take significantly longer than automatic processes, such as habits, the second term captures that due to interference between overlapping pathways the cost of a control signal increases with its intensity [13] even when control intensity accelerates the decision process [11,50,51]. In many real-world scenarios and some experiments, the opportunity cost is time-varying. This can be incorporated into our model by adding a learning mechanism that estimates *ω* from experience [52,53]. Following [46], we model the intrinsic cost of control as the implementation cost

$$cost(c)=\text{exp}(a_{i}\cdot ||c||+b_{i}),$$ (15)

where *c* is the control signal, *a*_*i*_ specifies how rapidly control cost increases with control intensity, and *b*_*i*_ determines the lowest possible cost. The monotonic increase of control cost with control signal intensity expressed by this equation models the fact that the more intensely you focus on one process, say color-naming, the less you are able to do other valuable things, such as verbal reasoning. This cognitive opportunity cost of control is a consequence of overlap between neural pathways serving different functions [13,54,55]. We do not assume any reconfiguration costs [46] but our framework can be easily extended to include them.

In all of our simulations, the number of samples drawn from the posterior distribution on the weights was *k* = 2. For simplicity, we modeled control allocation in each trial of the Stroop and Flanker tasks simulated below as an independent, non-sequential, metacognitive control problem. The opportunity cost of time (*ω* in Eq 14) was set to $8/h [cf. 51]. Model parameters were fitted by maximum likelihood estimation using Bayesian optimization [56]. The drift rates of the controlled and the automatic process (Eq 12) were determined from people’s response times on neutral trials. The model’s prior precision on the weights was set to assign 95% confidence to the EVOC of a stimulus lying between the equivalent of ±5 cents per second.

**Fig 2:**
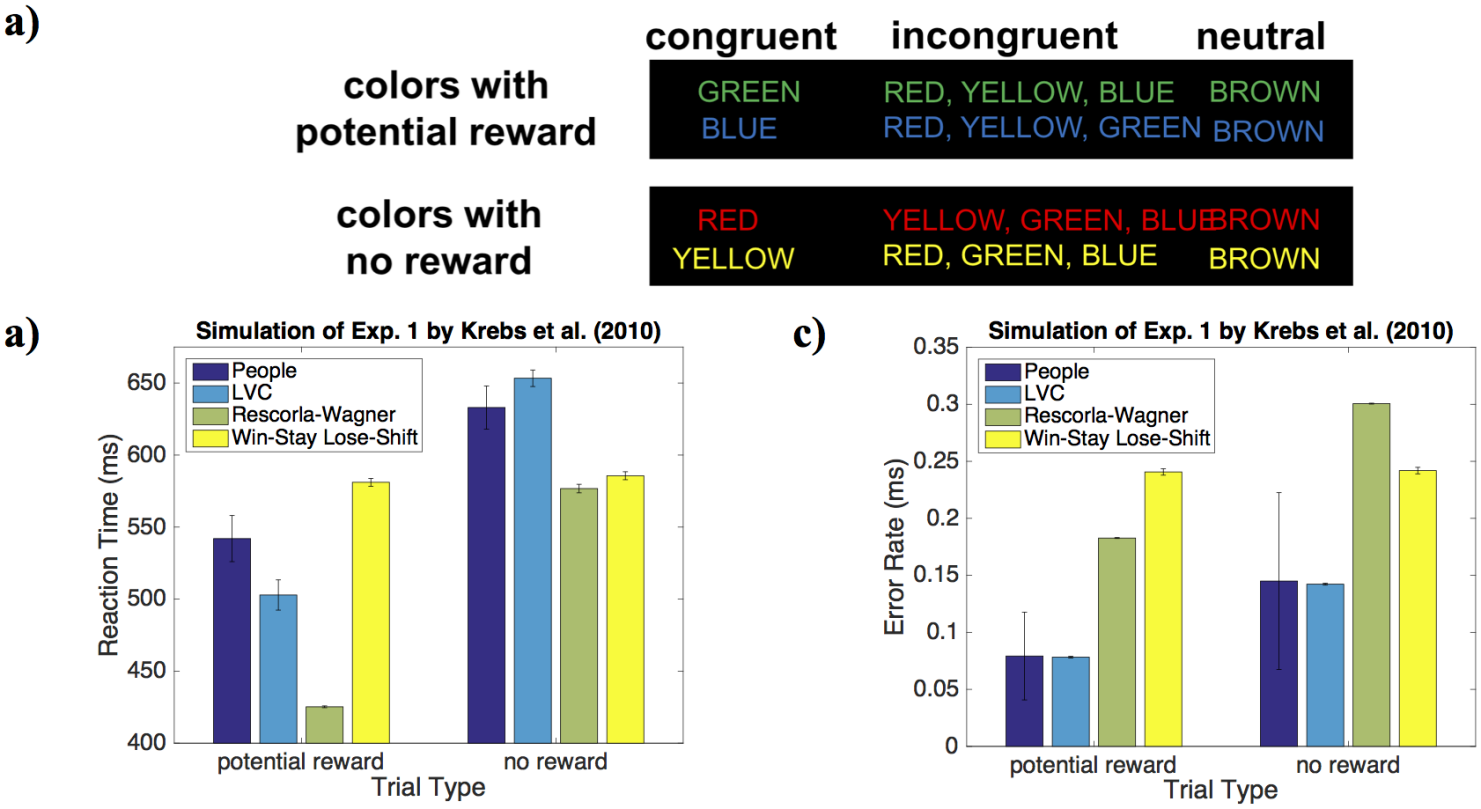
LVOC model captures that in the paradigm by Krebs et al. (a) People learn to exert more cognitive control on stimuli whose features predict that performance will be rewarded which manifests in faster responses (b) and fewer errors (c).

In the color-word Stroop task by Krebs et al. [11] the participant’s task was to name the font color of a series of color words which were either congruent or incongruent with the word itself (Fig 2a). For two of the four colors, giving the correct response yielded a monetary reward whereas responses to other two colors were never rewarded. Our simulation of this experiment assumed that people represent each stimulus by a list of binary features that encode the presence of each possible color and each possible word independently but do not encode their combinations. To capture the contribution of the experiment’s financial incentives for correct responses, we assumed that the utility *u*(*X*) in Eq 2 is the sum of the financial reward and the intrinsic utility of getting it right [57], that is

$$u(correct)=r_{external}+r_{intrinsic},$$ (16)

$$u(wrong)=-(r_{external}+r_{intrinsic}),$$ (17)

where the monetary reward *r*_external_ was 10 cents on rewarded trials and zero otherwise. The non-decision time was set to 300ms. The implementation cost parameters (*a*_*i*_ and *b*_*i*_ in Eq 15), the probability of accidental response flips (*p*_flip_), the intrinsic reward *r*_intrinsic_ of responding correctly (Eqs 16 and 17), and the noise parameter *σ* of the drift diffusion model (Eq 12) were fit to the empirical data shown in Fig 2b and 2c. To enable a fair comparison, we gave the associative learning model and the Win-Stay Lose-Shift model degrees of freedom similar to those of the LVOC model by adding parameters for the intrinsic reward of being correct, the probability of response error, and the noise of the drift-diffusion process. In addition, the Rescorla-Wagner model was equipped with a learning rate parameter. Each model was fitted using maximum-likelihood estimation using the Bayes adaptive direct search algorithm [58].

**Fig 3:**
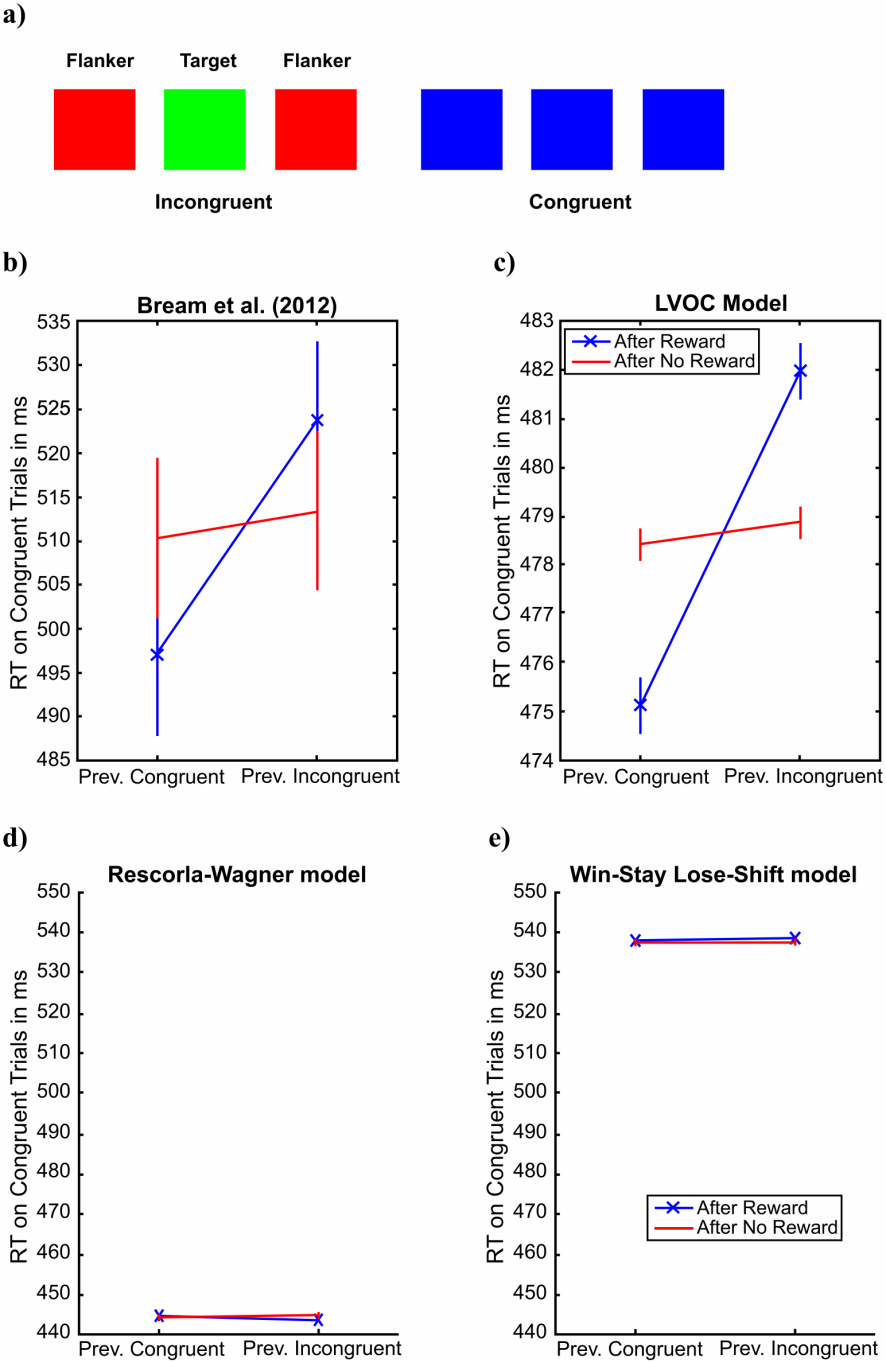
Metacognitive reinforcement learning captures the effect of reward on learning from experienced conflict observed by Braem et al. (2012). a) Illustration of the Flanker task by Braem et al. (2012). b) Human data by Braem et al. (2012). c) Fit of LVOC model. d) Fit of Rescorla-Wagner model. e) Fit of Win-Stay Lose-Shift model.

In the Flanker task by Braem et al. [10], participants were instructed to name the color of a central square (the *target*) flanked by two other squares (*distractors*) whose color was either the same as the color of the target (congruent trials) or different from it (incongruent trials) (Fig 3a). On a random 25% of the trials, responding correctly was rewarded and on the other 75% of the trials it was not. Our simulation assumed that people predict the EVOC from two features that encode the presence of conflict and congruency respectively: The conflict feature was +1 when the flankers and the target differed in color and zero otherwise. Conversely, the value of the congruency feature was +1 when the flankers had the same color as the target and zero else. To capture that people exert more cognitive control when they detect conflict [59], the prior mean on these weights was +1 for the interaction between control signal intensity and incongruence and −1 for the interaction between control signal intensity and congruence. Providing our model with these features instantiates our assumption that in the Flanker task perception is easy but response inhibition can be challenging. In other words, our model assumes that errors in the Flanker arise from the failure to translate three correct percepts into one correct response by inhibiting the automatic responses to the other two. Furthermore, the incongruency feature can also be interpreted as a proxy for the resulting response conflict that is widely assumed to drive the within-trial adjustment of control signals in the Flanker task [59].

Our simulation assumed that people only learn on trials with feedback. The effect of control was modelled as inhibiting the interference from the flankers according to

$$d=d_{target}+(1-c)\cdot C\cdot d_{flankers},$$ (18)

where *C* = 1 if the distractors are congruent and *C* = −1 when they are incongruent. The drift rates for accumulating information from the target (*d*_target_) and the distractors (*d*_flankers_) were assumed to be identical. Their value was fit to the response time for color naming on rewarded neutral trials reported in [11], and the non-decision time was 300ms. The perceived reward value of the positive feedback was determined by distributing the prize for high performance (EUR 10) over the 168 rewarded trials of the experiment (*z* = 7.5 US cents per correct response). Braem et al. [10] found that the effect of reward increased with people’s reward sensitivity. To capture individual differences in reward sensitivity, we modelled people’s subjective utility by

$$u(correct)=z^{\alpha }+r_{intrinsic},$$ (19)

$$u(wrong)=-r_{intrinsic},$$ (20)

where *z* ≥ 0 is the payoff and *α* ∈ [0,1] is the reward sensitivity. The reward sensitivity was set to 1, and the intrinsic reward of being correct (*r*_*i*ntrinsic_), the standard deviation of the noise (*σ*), the threshold of the drift-diffusion model (*θ*), the implementation cost parameters (*a*_*i*_, *b*_*i*_) were fit to the effects of reward on the reaction times on congruent trials (Fig 3c), the average reaction time, and the effect of reward sensitivity on conflict adaptation reported by [10]. The probability of accidentally giving the opposite of the intended response was set to zero.

To enable a fair comparison between LVOC model and the two simpler models, we equipped the associative learning model and the Win-Stay Lose-Shift model with the same assumptions and degrees of freedom as the LVOC model. Equivalently to the LVOC model of this task, they included a bias against exerting control was instantiated by an association of -1 between either stimulus feature and control exertion. The effect of control was modeled using the same drift-diffusion model with same set of free parameters, and like the LVOC model they also included a free parameter for the intrinsic reward of being correct and the probability of response error. Furthermore, these models included a free parameter for the cost of exerting control that is equivalent to two parameters of the LVOC model’s implementation and reconfiguration cost parameters, because their control signal was either 1 or 0. Furthermore, the Rescorla-Wagner model included an additional parameter for its learning rate, giving it the same number of parameters as the LVOC model.

**Fig 4:**
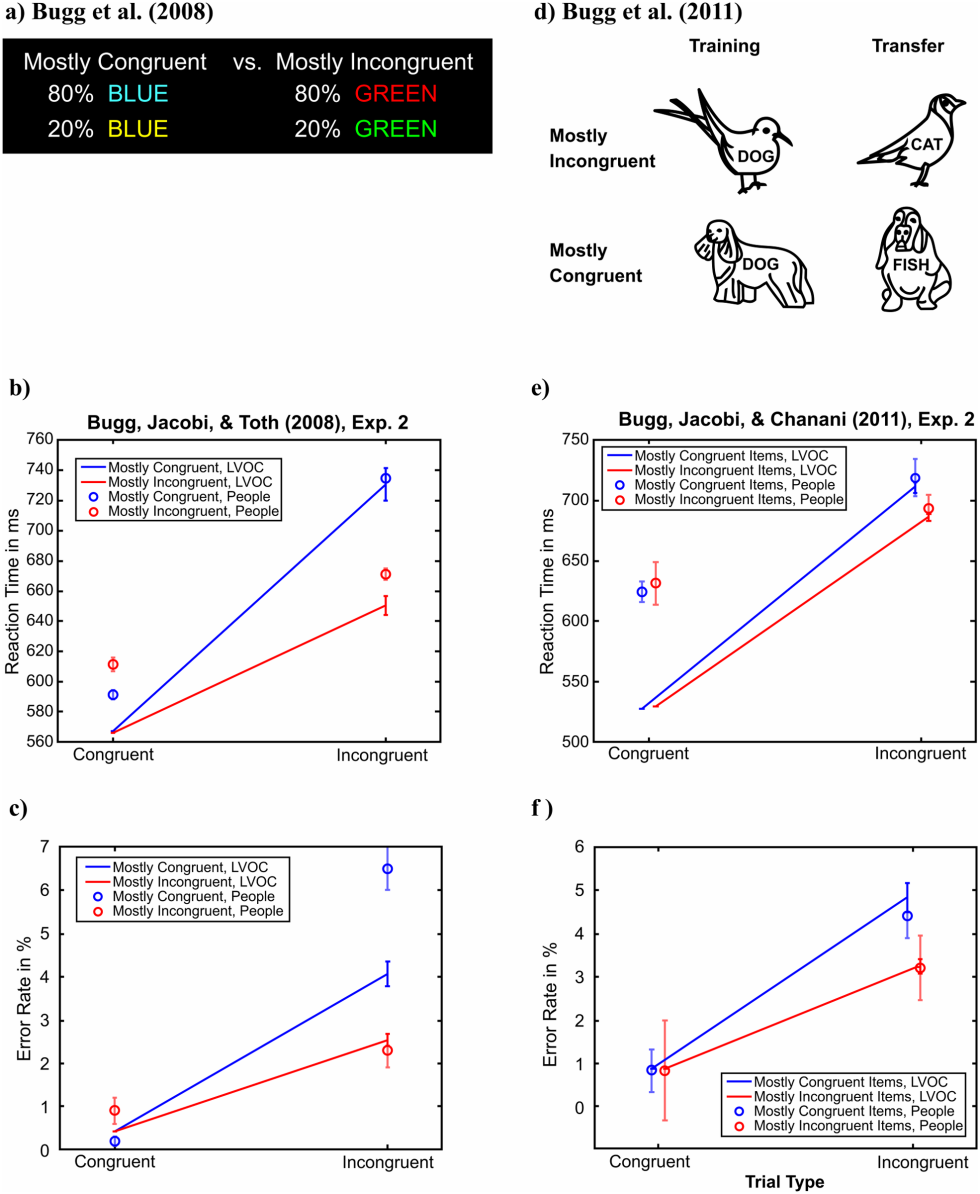
The LVOC model captures the finding that people learn to adjust their control intensity based on features that predict incongruence. a) Color-Word Stroop paradigm by Bugg et al. (2008). b-c) LVOC model captures that people learn to exploit features that predict incongruency to respond faster and more accurately on incongruent trial. d) Picture-Word Stroop paradigm by Bugg, Jacoby, and Chanani (2011). e-f) Just as human participants, the LVOC model responds more quickly and accurately to novel exemplars from animal categories that it previously learned to associate with more frequent incongruent trials.

Experiment 2 by Bugg et al. [8] asked participants to name the color of Stroop stimuli like those used by Krebs et al. [11]. Critically, some of the color words were printed in color that appeared on congruent trials 80% of the time whereas other color words were printed in a color that appeared on incongruent trials 80% of the time (Fig 4a). Each word was written either in cursive or standard font. We modeled the stimuli by four binary features indicating the presence of each of the four possible words (1 if the feature is present and 0 otherwise), and a fifth feature indicating the font type (0 for regular and 1 for cursive). The non-decision time was set to 400ms. Since there were no external rewards for good performance, the utility of correct/incorrect responses was ± *r*_intrinsic_. The implementation cost parameters (*a*_*i*_ and *b*_*i*_), the probability of accidental response flips (*p*_flip_), the intrinsic reward of being correct (*r*_intrinsic_), and the standard deviation of the noise (*σ*) were fit to the empirical data shown in Fig 4b and 4c. Given these parameters, the drift rate for color naming and reading where determined to match the reaction times on unrewarded neutral trials reported in [11].

Finally, Bugg et al. [9] presented their participants with pictures of animals overlaid by animal names Fig 4d). The participants’ task was to name the animal shown in the picture. Critically, for some animals, the picture and the word were usually congruent whereas for other animals the picture and the word were usually incongruent. The training phase was followed by a test phase that used novel pictures of the same animal species. We modelled this picture-word Stroop by representing each stimulus by a vector of binary indicator variables. Concretely, our representation assumed one binary indicator variable for each word (i.e., BIRD, DOG, CAT, FISH) and one indicator variable for each image category (i.e., bird, dog, cat, fish). The non-decision time was set to 400ms. The implementation cost parameters (*a*_*i*_ and *b*_*i*_), the intrinsic reward of being correct (*r*_intrinsic_), the standard deviation of the noise (*σ*), and the probability of accidental response flips (*p*_flip_) were fit to the empirical data shown in Fig 4e and 4f. Given these parameters, the drift rate for word reading was fit as above and the drift rate for picture naming was fit to a response time of 750ms.

## Results

We found that our model correctly predicted the learning effects observed in five different cognitive control experiments by virtue of its fundamental assumption that people reinforcement-learn to predict the value of potential control signals and control signal intensities from situational features (see Table 1). The following sections describe these findings in detail.

### Plasticity of attentional control in visual search

Lin et al. [17] had participants perform a visual search task for which the target of attention could either be predicted (training and predictable test trials) or not (unpredictable test trials) (Fig 1a). For this task, given its core reinforcement learning assumption (Table 1), the LVOC model predicts that 1) people should learn to attend to the circle with the predictive color and thus become faster at finding the target over the course of training, 2) continue to use the learned attentional control strategy in the test block and hence be significantly slower when the target appears in a circle of a different color during the test block, and 3) gradually unlearn their attentional bias during the test block (Fig 1c). As shown Fig 1b, all three predictions were confirmed by Lin and colleagues [17].

We compared the performance of LVOC to two plausible alternative models of these control adjustments: a Win-Stay Lose-Shift model and a simple associative learning model based on the Rescorla-Wagner learning rule. We found that the Win-Stay Lose-Shift model failed to capture that people’s performance improved gradually during training, and it also failed to capture the difference between people’s response times to predicted versus unpredicted target locations in the test block (see Fig 1d). As Fig 1e shows, the fit of the associative learning model (estimated learning rate: 0.0927) captures that after learning to exploit the predictive regularity in the training block participants were significantly slower in the test block. However, this simple model predicted significantly less learning induced improvement and significantly slower reaction times than was evident from the data by [17]. A quantitative model comparisons using the Bayesian Information Criterion [60,61] provided very strong evidence that the LVOC model explains the data by [17] better than the Rescorla-Wagner model or the Win-Stay Lose-Shift model (BIC_LVOC_ = 1817.8, BIC_RW_ = 9763.2, BIC_WSLS_ = 3449.9). This reflects that our model was able to accurately predict the data from [17] without any free parameters being fitted to those data. In conclusion, findings suggest that the LVOC model correctly predicted essential learning effects observed by [17] and explains these data significantly better than a simple associative learning model and a Win-Stay Lose-Shift model.

To more accurately capture both the slow improvement in the training block and the rapid unlearning in the test block simultaneously, the LVOC model could be extended by including a mechanism that discounts what has been learned or increases the learning rate when a change is detected [62,63]. Next, we evaluate the LVOC model against empirical data on the plasticity of inhibitory control.

### Plasticity of Inhibitory control

We found that our model can capture reward-driven learning effects in Stroop and Flanker tasks, as well as how people learn to adjust their control allocation based on features that predict incongruence and the transfer of these learning effects to novel stimuli. In each case, the LVOC model captured the empirical phenomenon more accurately than either a simple Win-Stay Lose-Shift model or a simple associative learning model. The following two sections present these results in turn.

### Reward-driven plasticity in interference control

#### People learn to allocate more control on rewarded trials

To determine whether our integrated theory captures the reward-modulated plasticity of cognitive control specification, we used the LVOC model to simulate two sets of experiments that examined the influences of reward on cognitive control. Krebs et al. [11] found that participants performing a color-word Stroop task learned to respond faster and more accurately to incongruently colored color words when their color predicted that performance would be rewarded than when the color predicted that performance would not be rewarded. We found that our model can capture these effects with reasonable parameter values (see Table 2). Fig 2 shows that our model captures Krebs et al.’s finding that people learn to exert more control on trials with rewarded colors than on trials with unrewarded colors even though they were interspersed within the same block. Concretely, our model captured that people become faster (691 ± 8ms vs. 541 ± 7ms; *t*(959998) = −14.6, *p* < 10^−15^) and more accurate (11.8 ± 0.03% errors vs. 4.9 ± 0.02% errors; *t*(959998) = −164.7, *p* < 10^−15^) when the color of the word is associated with reward. Critically, the qualitative effects observed in this experiment follow logically from the core assumption of the LVOC model (see Table 1).

**Table 2:** Model parameters used in the simulations of empirical findings.

	*a*_*i*_	*b*_*i*_	*θ*	*σ*	*r*_intrinsic_	*p*_flip_
Krebs, et al. (2010)	1.60	−0.01	3	0.05	1.60¢	3.5%
Braem et al. (2012)	4.17	−2	2.75	5	4.17¢	0.8%
Bugg et al. (2008)	1.95	−2.1	2.65	3.01	3.89¢	0.4%
Bugg et al. (2011)	5	−2	2.75	3	18.00¢	0.8%

We compared the LVOC model’s performance to that of an associative learning model with equivalent parameters (see Models); the maximum likelihood estimates of these parameters were *α* = 0.0447 for the learning rate, *r*_intrinsic_ = 0.1811 for the intrinsic reward, *σ*_*ε*_ = 0.1525 for the noise of the drift-diffusion process, and *p*_error_ = 0.1799. While the Rescorla-Wagner model was able to qualitatively capture the effect of potential reward on reaction time and error rate, its quantitative fit was far worse than the fit of the LVOC model (see Fig 2b and 2c); thus, a quantitative model comparison controlling for the number of parameters provided very strong evidence for the LVOC model over the Rescorla-Wagner model (BIC_LVOC_ = 45.3 vs. BIC_RW_ = 1333.9). We also fitted the Win-Stay Lose-Shift model and its parameter estimates were *r*_intrinsic_ = 0 for the intrinsic reward, *σ*_*ε*_ = 0 for the noise of the drift-diffusion process, and *p*_error_ = 0.07). We found that the WSLS model was unable to capture the effect of reward on response times and error rates (see Fig 2b and 2c) because its control signals are uninformed by the stimulus presented on the current trial. Consequently, a formal model comparison provided strong evidence for the LVOC model over the Win-Stay Lose-Shift model (BIC_LVOC_ = 45.3 vs. BIC_WSLS_ = 2454.8).

#### Reward accelerates trial-by-trial learning of how to allocate control

Braem et al. [10] found that participants in their Flanker task allocated more cognitive control after rewarded incongruent trials than after rewarded congruent trials or unrewarded trials. As Fig 3b shows, the LVOC model can capture this reward-induced conflict-adaptation effect with a plausible set of parameters (see Table 2). Our model correctly predicted that people’s responses on congruent trials are faster when they are preceded by rewarded congruent trials than when they are preceded by rewarded incongruent trials. The predicted difference (7 ms) was smaller than the empirically observed difference (27 ms) but it was statistically significant (*t*(99) = 37.99, *p* < 10^−15^). According to our model, people learn to exert more control on incongruent trials than on congruent trials. Furthermore, being rewarded for exerting a low level of control reduces the control intensity on the subsequent trial, whereas being rewarded for exerting a high level of control increases the control intensity on the subsequent trial. Thus, our model predicts that control intensity should increase after rewarded incongruent trials but decrease after rewarded congruent trials. On congruent trials, more control leads to slower responses because it inhibits the facilitating signal from the flankers (Eq 18). This suggests that our model’s metacognitive reinforcement learning mechanism correctly predicts the findings of Braem et al. [10] (see Fig 3 and Table 1).

The LVOC model’s learning increases with the magnitude of the reward. Consequently, the LVOC model predicts that the effect shown in Fig 3b should increase with people’s reward sensitivity. Concretely, as we increased the reward sensitivity parameter *α* from 0 to 1, the predicted reward-driven effect of conflict monotonically increased from 0.6ms to 8.0ms (*t*(102) = 4.77, *p* < 0.0001). Consistent with this prediction, Braem and colleagues [10] found that the magnitude of reward-driven conflict adaptation effect increased with people’s reward sensitivity, suggesting that the reward experienced for exerting cognitive control was the driving force of their adjustments. The significant positive correlation between people’s reward sensitivity and the magnitude of their conflict adaptation effect reported by [10] confirms our model’s prediction. Our model captures all of these effects because it learns to predict the expected rewards and costs of exerting control from features of the situation and probabilistically chooses the control signal that achieves the best cost-benefit tradeoff.

We compared the LVOC’s fit to these behaviors with the associative learning and Win-Stay Lose-Shift models. Even though the associative learning model had the same number of parameters as the LVOC model, its fit was substantially worse than the fit of the LVOC model (mean squared errors: MSE_RW_ = 3.33 vs. MSE_LVOC_ = 1.35), and its best fit (Fig 3d) failed to capture the qualitative effect shown in Fig 3c. Finally, we evaluated a Win-Stay Lose-Shift model. This model was equipped with the same set of parameters as our Rescorla-Wagner model except for the learning rate parameter. We found that the Win-Stay Lose-Shift model was unable to capture the data by Braem et al. Fig 3e) because it stays with the controlled process forever once it has been rewarded for using it (MSE_WSLS_ = 19.4). Taken together with the previous results, this suggests that the simple mechanisms assumed by the associative learning model and the Win-Stay Lose-Shift model are insufficient to explain the complexity of cognitive control plasticity, but the LVOC model can capture it.

### Transfer of learning effects in interference control

The expected value of computation depends not only on the rewards for correct performance but also on the difficulty of the task. In easy situations, such as the congruent trials of the Stroop task, the automatic response can be as accurate, faster, and less costly than the controlled response. In cases like this, the expected value of exerting control is less than the EVOC of exerting no control. By contrast, in more challenging situations, such as incongruent Stroop trials, the controlled process is more accurate and therefore has a positive EVOC as long as accurate performance is sufficiently important. Therefore, on incongruent trials the expected value of control is larger than the EVOC of exerting no control. Our model thus learns to exert control on incongruent trials but not on congruent trials. Our model achieves this by learning to predict the EVOC from features of the stimuli. This predicts that people should learn to exert more control when they encounter a stimulus feature (such as a color or word) that is predictive of incongruence than when they encounter a feature that is predictive of congruence (see Table 1).

Consistent with our model’s predictions, Bugg and colleagues [8] found that people learn to exert more control in response to stimulus features that predict incongruence than stimulus features that predict congruence. Their participants performed a color-word Stroop task with four colors and their names printed either in cursive or regular font. Our model captured the effects of congruency-predictive features on control allocation with a plausible set of parameters (see Table 2). As shown in Fig 4a and 4b, the LVOC model predicted that responses should be faster (655 ± 9 ms vs. 722 ± 11 ms; *t*(49) = 5.39, *p* < 0.0001) and more accurate (2.85 ± 0.2% errors vs. 4.3 ± 0.3% errors; *t*(49) = 5.01, *p* < 0.0001) on incongruent trials if the word was predictive of incongruence than when it was not. To their surprise, Bugg and colleagues observed that adding an additional feature (font) that conveyed the same information about congruence as the color, did not enhance learning. This is exactly what our model predicted because the presence of a second predictive feature reduces the evidence for the predictive power of the first one and vice versa–this is directly analogous to a phenomenon from the Pavlovian literature known as blocking, whereby an animal fails to learn an association between a stimulus and an outcome that is already perfectly predicted by a second stimulus [64].

Since our model learns about the predictive relationship between features and the EVOC, it predicts that all learning effects should transfer to novel stimuli that share the features that were predictive of the expected value of control in the training trials (see Table 1). A separate study by Bugg and colleagues [9] confirmed this prediction. They trained participants in a picture-word Stroop task to associate particular images of certain categories (e.g., cats and dogs) with incongruence and associated particular images of other categories (e.g., fish and birds) with congruence. As expected, participants learned to exert more control when viewing the stimuli associated with incongruence. More importantly, these participants also exerted more control when tested on *novel* instances of the category associated with incongruence (e.g., cats) than on novel instances of the category associated with congruence (e.g., fish). This finding provides strong evidence for the feature-based learning mechanism that is at the core of our model of the plasticity of cognitive control and is entirely accounted for by our model. As shown in Fig 4e and 4f, our model correctly predicted the positive and the negative transfer effects reported by [9] with reasonable parameters (see Table 2): The model’s responses were faster (709 ± 3 ms vs. 685 ± 2 ms; *t*(99) = −8.13, *p* < 0.0001) and more accurate (4.8 ± 0.3% errors vs. 3.2 ± 0.1% errors; *t*(99) = −5.06, *p* < 0.0001) on incongruent trials if the word was predictive of incongruence than when it was not (positive transfer). Conversely, on congruent trials, the predicted responses were slightly slower when the features wrongly predicted incongruence (527 ± 0.2ms vs. 530 ± 0.1ms, *t*(99) = 9.28, *p* < 0.0001; negative transfer).

## Discussion

Building on previous work modeling the specification of cognitive control in terms of meta-decision making [12–14,29,33,65,66] and reinforcement learning [33,34,67–69], we have illustrated that at least some of the functions of cognitive control can be characterized using the formal framework of rational metareasoning [26] and meta-level Markov decision processes [27]. Concretely, modeling the function of cognitive control as a meta-level MDP allowed us to derive the first formal computational model of how people learn to specify continuous control signals and how these learning effects transfer to novel situations. This model provides a unifying explanation for how people learn where to attend, the interacting effects of reward and incongruence on interference control, and their transfer to novel stimuli.

Our simulations of learning in Stroop and Flanker paradigms illustrate that the LVOC model can account for people’s ability to learn when and how intensely to engage controlled processing and inhibit automatic processing. We further found that the LVOC model correctly predicted the learning curve in the visual attention experiment by Lin et al. [17] without any free parameters. Critically, all of our model’s qualitative predictions follow directly from our theory’s core assumption that people reinforcement-learn to predict the value of alternative control signals and control signal intensities from stimulus features (see Table 1). None of our model’s auxiliary assumptions about the cost of control, the reward for being correct, the drift-diffusion model, the details of learning and control signal selection, and the corresponding parameters summarized in Table 2 are necessary to derive these qualitative predictions; instead they only serve to increase the quantitative accuracy of those predictions.

While the LVOC model is more complex than basic associative learning and the Win-Stay Lose-Shift mechanism, neither of these simpler models was able to capture human learning in the simulated visual search, Stroop, and Flanker paradigms. This suggests that the complexity of the LVOC model may be currently warranted to capture how people learn when to exert how much cognitive control. Furthermore, the LVOC model’s sophistication may be necessary to explain more complex phenomena such as how people learn to orchestrate their thoughts to solve complex problems and acquire sophisticated cognitive strategies. Recent work has indeed shown that the learning mechanism instantiated by the LVOC model can also capture aspects of how people learn how to plan [70] and to flexibly and adaptively choose between alternative cognitive strategies [53]. Testing whether people learn to select sequences of control signals in the way predicted by our model is an interesting direction for future research.

### The LVOC model integrates control specification and strategy selection learning

The model developed in this article builds on two previous theories: the EVC theory, which offered a normative account of control specification [7], and the rational metareasoning theory of strategy selection [53], which suggested that people acquire the capacity to select heuristics adaptively by learning a predictive model of the execution time and accuracy of those heuristics. The LVOC model synergistically integrates these two theories: it augments the EVC theory with the metacognitive learning and prediction mechanisms identified by [53], and it augments rational metareasoning models of strategy selection with the capacity to specify continuous control signals that gradually adjust parameters of the controlled process (see S2 Text).

### Empirical predictions

All else being equal, the proposed learning rules (see Eq 7, S1 Text Equations 1–7, and S3 Text Equations 13–14) predict that people’s propensity to exert cognitive control should increase when the controlled process was less costly (e.g., faster) or generated more reward than expected [19]. The experience that less controlled (more automatic) processing was more costly or less rewarding than expected should also increase our propensity to exert cognitive control [71–73]. Conversely, if a controlled process performed worse than expected or if an automatic process performed better than expected, people’s propensity to exert cognitive control should decrease [74].

At a more detailed level, our theory predicts that the influence of environmental features on control allocation generalizes across contexts, to the extent that their features are similar. Thus, adding or removing features to the internal predictive model of the EVOC should have a profound effect on the degree to which observed performance of the controlled process in Context A changes people’s propensity to select it in Context B, and vice versa. This mechanism can account for empirical evidence that suggests a role for feature-binding in mechanisms of task switching [75–78]. These studies suggest that participants associate the task that they perform on a stimulus with the features of that stimulus. Once they are asked to engage in a new task on that stimulus, the old (associated) task interferes, leading to switch costs.

Furthermore, our theory predicts that increasing the rewards and punishments for the outcomes of the controlled or automatic processes should increase the speed with which people’s control allocation adapts to new task requirements, because the resulting weight updates will be larger; this becomes especially apparent when the updates are rewritten in terms of prediction errors (see S3 Text, Eqs 1 and 2). Finally, when the assumptions of the internal model are met and its features distinguish between the situations in which each controlled process performs best, then control signal selection should become increasingly more adaptive over time [79,80]. But in situations where the internal model’s assumptions are violated, for instance because the value of control is not additive and linear in the features, then the control system’s plasticity mechanisms may become maladaptive.

This prediction has been confirmed in a recent experiment with a novel color-word Stroop paradigm comprising two association phases and a test phase [81]. In the first association phase, participants learned that color naming was rewarded for certain colors whereas word reading was rewarded for the other colors. In the second association phase, participants learned that color-naming was rewarded for certain words whereas word-reading was rewarded for other words. Critically, in the test phase, naming the color was rewarded if either the word or the color had been associated with color naming (SINGLE trials); but when both the color and the word were associated with color naming then participants had to instead read the word (BOTH trials). This non-linear relationship between stimulus-features and control demands caused mal-transfer from SINGLE trials to BOTH trials that significantly interfered with participants’ performance (resulting in participants incorrectly engaging in color-naming, the more control-demanding task which in that context was *also* less rewarding). The LVOC model may thus be able to explain the puzzling phenomenon that people sometimes overexert cognitive control even when it hurts their performance. For instance, if your past experience has taught you to choose your words very carefully on a certain topic then receiving an email on that topic might compel you to mentally compose a perfect response even when you would be better off thinking about how to open the talk you have to deliver in 5 minutes.

According to the LVOC model, control allocation is a process of continuing gradual adjustment (Eq 13). This means that the control intensity for a new situation starts out with the control intensity from the previous situation and is then gradually adjusted towards its optimal value—just like in anchoring-and-adjustment [48,82]. This might provide a mechanism for commonly observed phenomena associated with task set inertia and switch costs [47]. Since control adjustment takes time, this mechanism predicts that increased time pressure could potentially lead to decreased control adjustment, thereby biasing people’s control allocation to its value on the previous trial and thus decreasing their cognitive flexibility. Finally, thinking about the neural implementation of the LVOC model leads to additional neural predictions as detailed in the S3 Text.

### Avenues for future research

We view rational metareasoning as a general theoretical framework for modeling the allocation and plasticity of cognitive control. As such, it could be used to develop unifying models of different manifestations of cognitive control, such as attention, response inhibition, and cognitive flexibility. Furthermore, rational metareasoning can also be used to connect existing models of cognitive control [6,7,32–34,46,65,66,79,80,83,84]. Interpreting previously proposed mechanisms of control allocation as approximations to rational metareasoning and considering how else rational metareasoning could be approximated might facilitate the systematic evaluation of alternative representations and computational mechanisms and inspire new models. While our computational explorations have focused on which control signal the cognitive control system should select, future work might also shed light on how the cognitive control system monitors the state of the controlled system by viewing the problem solved by the cognitive control system as a partially observable MDP. Concretely, the function of cognitive monitoring could be formulated as a meta-level MDP whose computational actions include sensing operations that update the cognitive control system’s beliefs about the state of the monitored system.

Future work should further evaluate the proposed computational mechanism and its neural implementation by performing quantitative model comparisons against simpler models across a wider range of cognitive control phenomena. This line of work should also evaluate the performance of the proposed metacognitive learning mechanism and evaluate it against alternative mechanisms (e.g., temporal difference learning mechanisms with eligibility traces [30]).

Another interesting direction will be to use the learning models to investigate the plasticity of people’s cognitive control skills. We are optimistic that this line of work will lead to better quantitative models of control plasticity that can be used to develop interventions to improve people’s executive functions via a combination of cognitive training and augmenting environments where people’s automatic responses are maladaptive with cues that prime them to employ an appropriate control signal. In addition, future work may also explore model-based metacognitive reinforcement learning [85] as a model of the plasticity of cognitive control specification. Model-based hierarchical reinforcement learning approaches [37], such as option models [38], could be used to integrate the learning mechanisms for the value of individual control signals with the strategy selection model to provide an account of how the brain discovers control strategies. This might explain how people learn to adaptively coordinate their thoughts and actions to pursue increasingly more challenging goals over increasingly longer periods of time.

Finally, the rational metareasoning framework can also be used to model how people reason about the costs and benefits of exerting mental effort and to delineate self-control failure from rational resource-preservation through a normative account of effort avoidance [13,86].

### Conclusion

Our simulation results suggested that the LVOC model provides a promising step towards a mathematical theory of cognitive plasticity that can serve as a scientific foundation for designing cognitive training programs to improve people’s executive functions. This illustrates the utility of formalizing the function of cognitive control in terms of rational metareasoning. Rational metareasoning provides a unifying framework for modeling executive functions, and thus opens up exciting avenues for future research. We are optimistic that the connection between executive functions and metareasoning will channel a flow of useful models and productive ideas from artificial intelligence and machine learning into the neuroscience and psychology of cognitive control.

## Supporting information

S1 Text Mathematical details of the LVC model’s learning mechanism. (DOCX)

S2 Text Rational metareasoning unifies the EVC theory with the rational metareasoning theory of strategy selection. (DOCX)

S3 Text Speculations about how the learning mechanism postulated by the LVC model might be implemented in the brain. (DOCX)
