# Supplementary material for: Rational metareasoning and the plasticity of cognitive control
Source: PLoS Comput Biol. 2018 Apr 25;14(4):e1006043. doi: 10.1371/journal.pcbi.1006043 (PMC5937797; doi:10.1371/journal.pcbi.1006043)
Supplement: S1 Text — (DOCX) [file pcbi.1006043.s001.docx]

# Mathematical details of the LVC model’s learning mechanism

The model learns from the observed EVOC proxy

$$\tilde{V}\left( s,\boldsymbol{c}_{t} \right)=r+\max_{o} \hat{q}_{t}\left( s',\boldsymbol{c}_{t};\boldsymbol{w} \right)-\omega\cdot T-\mathrm{cost}(\boldsymbol{c}_{t}), (1)$$

which integrates the reward $r$ with the predicted value of the resulting state $s'$, the control signal $\boldsymbol{c}_{t}$ at time $t$, the time cost $\omega\cdot T$ (where $T$ is the response time and $\omega$ is the agent’s reward rate). The learning mechanism assumes that the VOC proxy is a noisy instantiation of the true VOC, that is

$$\tilde{V}\left( s,\boldsymbol{c} \right)=Q_{t}^{\star}\left( s,\boldsymbol{c} \right)+\varepsilon, \varepsilon\sim\mathcal{N}\left( 0,\sigma_{\varepsilon} \right), (2)$$

where $Q_{t}^{\star}\left( s,\boldsymbol{c} \right)$ is the expected sum of rewards that the optimal control strategy would obtain after selecting control signal $\boldsymbol{c}$ in belief-state $s$. The likelihood function of the observed VOC proxy given the weights is the Gaussian

$$P\left( \tilde{V}\left( s,\boldsymbol{c} \right) | s,\boldsymbol{c},\boldsymbol{w} \right)\mathcal{=N}\left( Q_{t}^{\star}(s,c),\sigma_{\varepsilon} \right). (3)$$

The prior on the weights is a Gaussian shrinkage prior, that is

$$P\left( \boldsymbol{w} \right)\mathcal{=N}\left( \boldsymbol{\mu}_{\boldsymbol{0}}\boldsymbol{=0},\Lambda_{0}=\mathbf{I}_{n} \right), (4)$$

where $\mathbf{I}_{n}$ is the $n\times n$ identity matrix and $n$ is the total number of weights. Therefore, the posterior on the weights given one or more observations $o_{i}=\left( s_{i},c_{i},\tilde{V}_{i} \right)$ is given by

$$P\left( \boldsymbol{w|o} \right)\mathcal{=N}\left( \boldsymbol{\mu}_{\boldsymbol{n}},\Lambda_{n} \right), (5)$$

with

$$\Lambda_{n}=\boldsymbol{X}^{t}\boldsymbol{X}+\Lambda_{0} (6)$$

$$\boldsymbol{\mu}_{\boldsymbol{n}}\boldsymbol{=}\Lambda_{n}^{-1}\cdot\left( \Lambda_{0}\mu_{0}+X^{t}\boldsymbol{y} \right), (7)$$

where $\boldsymbol{y=}\left( \tilde{V}_{1},\cdots,\tilde{V}_{t} \right)^{\boldsymbol{t}}$**.** The matrix $\boldsymbol{X}$ contains the values of the VOC predictors in Equation 6 of the Main Text (offset, features, control intensities, interaction terms, and control costs) and its rows correspond to different trials.
