# Supplementary material for: Rational metareasoning and the plasticity of cognitive control
Source: PLoS Comput Biol. 2018 Apr 25;14(4):e1006043. doi: 10.1371/journal.pcbi.1006043 (PMC5937797; doi:10.1371/journal.pcbi.1006043)
Supplement: S2 Text — (DOCX) [file pcbi.1006043.s002.docx]

# Rational metareasoning unifies the EVC theory with the VOC theory

The Expected Value of Control (EVC) theory [1] and the rational metareasoning theory of strategy selection [2,3] both appeal to a tradeoff between expected performance and cost. According to the EVC theory, control signals $c$ are selected such as to maximize the expected value of control (EVC) given the expected state $s$ of the environment and the control signal $c$:

$$c^{\star}=\arg\max_{\mathbf{c}} \mathrm{EVC}\left( c,s \right), (8)$$

where the EVC of a control signal $c$ is the expected sum of the resulting immediate and (discounted) future rewards minus a cost ($\mathrm{cost}\left( c \right) )$that scales with the magnitude of the candidate control signal:

$$\mathrm{EVC}\left( c,s \right)=\sum_{k} P\left( x_{k} | c,s \right)\cdot u(x_{k})-\mathrm{cost}\left( c \right), (9)$$

where the outcome $x_{k}$ includes the next state and $u$ represents the utility of that outcome. The cost of control is composed of an implementation cost that increases with the intensity of the control signal and a reconfiguration cost that increases with the change from the previous signal [4].

The rational metareasoning theory of strategy selection holds that the brain should choose the cognitive strategy $c^{\star}$ with the best possible speed-accuracy tradeoff on the problem defined by the inputs $i$ as measured by the Value of Computation (VOC), that is

$$c^{\star}=\arg\max_{c} \mathrm{VOC}\left( c,i \right), \left( 10 \right)$$

$$\mathrm{VOC}\left( c,i \right)=\mathbb{E}\left[ U| i,c \right]-\omega\mathbb{\cdot E}\left[ T | i,c \right], \left( 11 \right)$$

where $U$ is the utility of the outcome obtained by strategy $c$, $\mathbb{E}\left[ T | i,c \right]$ is the strategy’s expected execution time, and $\omega$ is the agent’s opportunity cost per unit time. We therefore refer to this theory as the VOC theory for short. A key assumption of this theory is that the brain learns to predict the execution time and utility of applying a strategy $c$ by a linear combination of the features of the problem to be solved. This internal model is then used to predict each strategy’s execution time and the resulting gain in reward in order to choose the strategy with the highest predicted VOC.

Our rational metareasoning perspective on cognitive control unifies the EVC theory with the VOC theory: The EVC theory (Equations 8-9) can be seen as an instantiation of rational metareasoning for graded control signals that adjust parameters of the controlled process (Equations 1-4 of the Main Text). Concretely, in the EVC theory, $\mathbf{c}$ is a vector comprising the intensities $c_{1},c_{2},\cdots,c_{k}$ of $k$ distinct types of control signals (i.e., $\boldsymbol{c}=(c_{1},c_{2},\cdots,c_{k})$). Similarly, the VOC theory (Equations 10-11) instantiates the rational metareasoning framework for a specific class of control strategies (Equations 1-4 in the Main Text). Concretely, the VOC theory considers control strategies $o\in\mathcal{S}$ whose value is fully determined by the costs and rewards accrued during and immediately after their execution and thus independent of the next state. This reduces the optimal Q-function to $\mathbb{E}\left[ r\left( s,\pi\right) \right]$ plus a constant and the optimal strategy becomes

$$\pi^{\star}\left( s \right)=\arg\max_{o\in\mathcal{S}} \mathbb{E}\left[ r\left( s,o \right) \right]=\arg\max_{o\in\mathcal{S}} \mathbb{E}\left[ u\left( \text{X} \right)-cost\left( s,o \right) \right]=\arg\max_{o\in\mathcal{S}} \mathbb{E}\left[ U|s,o \right]\mathbb{-E}\left[ \mathrm{cost}\left( s,o \right)|s \right], (12)$$

where $U$ is the utility of the outcome $X$ obtained with the cognitive strategy $o$. Hence, in situations where the cost of the control strategy $o$ is the opportunity cost of its execution time ($\mathrm{cost}\left( s,o \right)=\omega\cdot T$)^[[1]](#footnote-1)^, the problem of optimal cognitive control simplifies into the strategy selection problem defined by the VOC theory (Equations 10-11):

$$\pi^{\star}\left( s \right)=\arg\max_{o\in\mathcal{S}} \mathbb{E}\left[ U|s,o \right]-\omega\cdot\mathbb{E}\left[ T|s,o \right]. (13)$$

Since the EVC theory and the VOC theory instantiate complementary aspects of rational metareasoning, they can be integrated into a more general theory of cognitive control specification that we call the Expected Value of Control (EVOC) theory of the function of cognitive control. This theory maintains that individual computations and cognitive strategies are selected according to a predictive model of their EVOC and can be specified through a vector comprising continuous and discrete control signals. The key advance of this theory is that it allows the value of control to be learned from experience, and the Learned Value of Control (LVOC) model specifies a concrete mechanism by which people might *learn* to select continuous control signals. The LVOC model thereby synergistically integrates the strengths of the EVC theory (i.e., continuous control signals) with the strengths of the rational metareasoning theory of strategy selection (i.e., learning and strategy selection).

# References

1. Shenhav A, Botvinick MM, Cohen J. The Expected Value of Control: An Integrative Theory of Anterior Cingulate Cortex Function. Neuron. Cell Press,; 2013;79: 217–240. doi:doi: 10.1016/j.neuron.2013.07.007

2. Lieder F, Plunkett D, Hamrick JB, Russell SJ, Hay NJ, Griffiths TL. Algorithm selection by rational metareasoning as a model of human strategy selection. In: Ghahramani Z, Welling M, Weinberger KQ, Cortes C, Lawrence ND, editors. Advances in Neural Information Processing Systems 27. Curran Associates, Inc.; 2014.

3. Lieder F, Griffiths TL. When to use which heuristic: A rational solution to the strategy selection problem. In: Noelle DC, Dale R, Warlaumont AS, Yoshimi J, Matlock T, Jennings CD, et al., editors. Proceedings of the 37th Annual Conference of the cognitive science society. Austin, TX: Cognitive Science Society; 2015.

4. Musslick S, Shenhav A, Botvinick MM, Cohen JD. A computational model of control allocation based on the Expected Value of Control. The 2nd Multidisciplinary Conference on Reinforcement Learning and Decision Making. 2015.

1. Note that the rational metareasoning framework is equally applicable to situations where the cost of control is primarily determined by other factors such as the intensity of the control signal that can be inversely related to reaction time. [↑](#footnote-ref-1)
