# Supplementary material for: Rational metareasoning and the plasticity of cognitive control
Source: PLoS Comput Biol. 2018 Apr 25;14(4):e1006043. doi: 10.1371/journal.pcbi.1006043 (PMC5937797; doi:10.1371/journal.pcbi.1006043)
Supplement: S3 Text — (DOCX) [file pcbi.1006043.s003.docx]

# Speculations about how the learning mechanism postulated by the LVC model might be implemented in the brain

The dorsal anterior cingulate cortex (dACC) has been found to be involved in virtually any task requiring cognitive control and has consequently been ascribed many different functions. The EVC theory [1] suggested that all of these findings reflect a single underlying function: the specification of cognitive control signals. It thereby provided a general account for the presence of a myriad of neural signals in this region related to potential states, control signals, and outcomes [1]; see also [2–6]. Building on these results, we postulate that the dACC plays a critical role in the plasticity of cognitive control specification. Our current account extends the EVC theory by providing a computational framework for the estimation of EVC/VOC that allows for a neurally plausible implementation (Figure 1). Concretely, the dACC might predict the expected value of alternative control signals within fractions of a second by the simple feed-forward network architecture shown in Figure 1. The inputs to the network convey the internal state of the controlled system along with representations of the context. A series of hidden layers extracts the features of the internal predictive model. The final layer maps the features onto the predicted value of control.


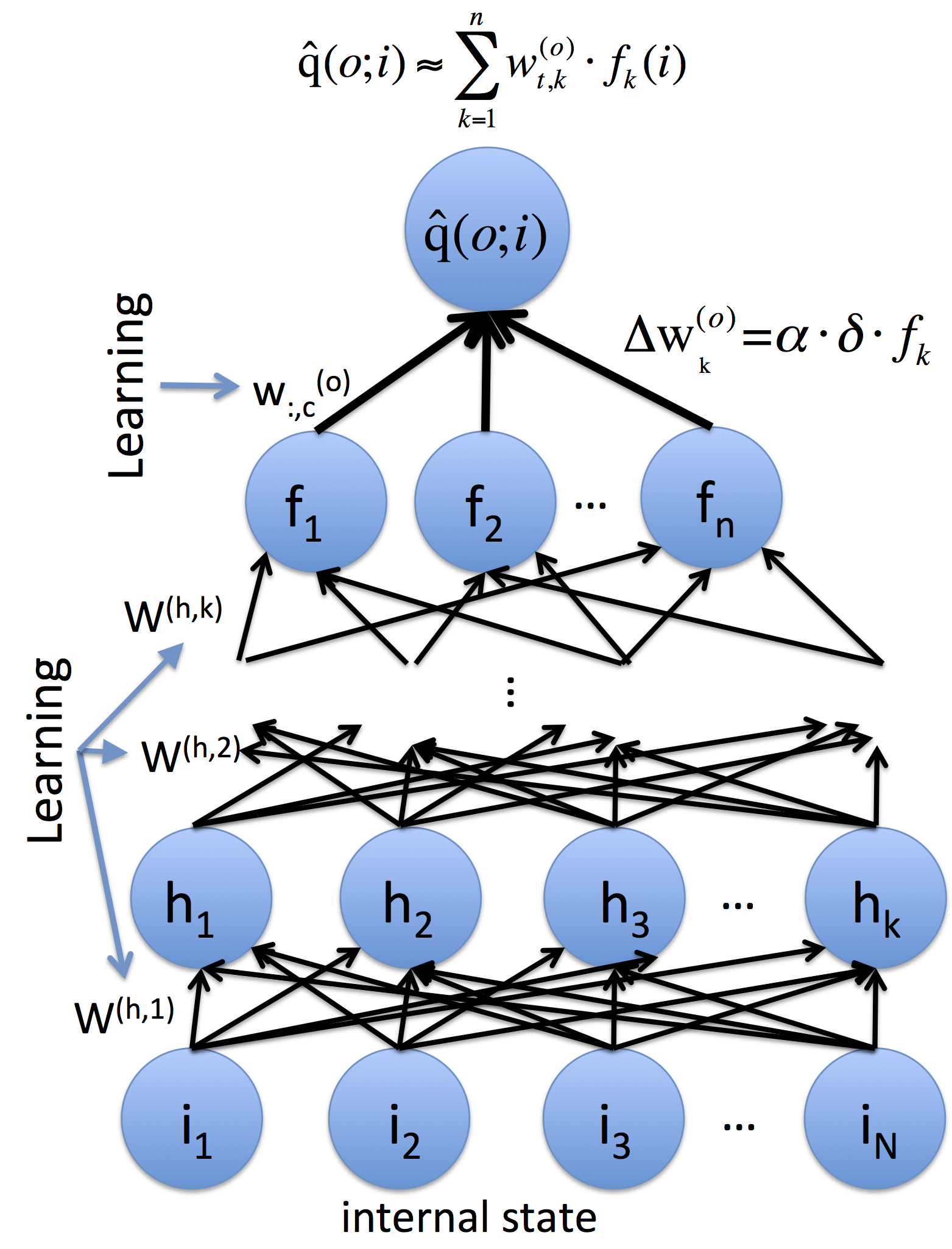


Figure 1: Our rational process model of strategy selection learning could be implemented in a simple feed-forward neural network.

Since the dACC receives dopaminergic prediction error signals from the ventral tegmental area [7], the proposed mechanism could plausibly be implemented through the dopaminergic modulation of synaptic plasticity in the dACC [8]. Concretely, the dACC might approximate the Bayes optimal learning of those weights, which we have assumed in the simulations above, by adjusting the weights $w$ according to a simple, biologically plausible learning rule based on the SARSA algorithm with function approximation [9]:

$$w_{t+1,k}=w_{t,k}+\alpha_{t}\cdot\delta\cdot f_{k}\left( s_{t},o_{t} \right), (1)$$

where $\alpha_{t}$ is a learning rate that decreases over time,

$$\delta=r\left( s_{t},o_{t} \right)+\hat{q}_{t}\left( s_{t+1},\pi_{t}(s_{t+1}) \right)-\hat{q}_{t}\left( s_{t},o_{t} \right), (2)$$

is the reward prediction error and $\pi_{t}$ is a policy based on $\hat{q}_{t}$; e.g. the softmax policy that chooses strategy $o$ with probability $\frac{\exp(\hat{q}_{t}(s_{t},o)/\tau)}{\sum_{o} \exp(\hat{q}_{t}(s_{t},o)/\tau}$ for some decision temperature $\tau>0$.

The metacognitive reward prediction error $\delta$ is similar to the reward prediction error conveyed by phasic dopamine that is elicited by a sensory cue that predicts future reward [10]. The main difference is that the information leading to the update of the reward prediction is provided by internal computation rather than perception of the external environment.

Our model can be used to constrain predictions for how these different types of neural signals are related to each other [11] and how they respond to changes in features of the task, such as incentives and cues that predict task difficulty, conflict, reward, performance, and progress [12]. Furthermore, the learning rules presented in Equations 1-7 of S1 Text and Equations 1-2 of this document predict how these neural responses should change with learning.

At a general level, our account predicts that synaptic plasticity between neurons representing contextual factors and dACC neurons representing the LVC of individual control signals should implement the learning rule specified in Equation 14. This equation predicts that the change in synaptic connectivity should be proportional to the product of the value of the feature times the metacognitive reward prediction error (Equation 15) that might be conveyed by dopamine. Thus, if the metacognitive reward prediction error is positive, then we should observe long-term potentiation but when it is negative, then we should see long-term depression. If the metacognitive reward prediction error was indeed conveyed by phasic dopamine then we would also expect to see spontaneous bursts of dopaminergic VTA neurons that reflect the outcomes of internal computations [3,13,14].

# References

1. Shenhav A, Botvinick MM, Cohen J. The Expected Value of Control: An Integrative Theory of Anterior Cingulate Cortex Function. Neuron. Cell Press,; 2013;79: 217–240. doi:doi: 10.1016/j.neuron.2013.07.007

2. Silvetti M, Alexander W, Verguts T, Brown JW. From conflict management to reward-based decision making: actors and critics in primate medial frontal cortex. Neurosci \& Biobehav Rev. Elsevier; 2014;46: 44–57.

3. Holroyd CB, McClure SM. Hierarchical control over effortful behavior by rodent medial frontal cortex: A computational model. Psychol Rev. American Psychological Association; 2015;122: 54.

4. Shackman AJ, Salomons T V, Slagter HA, Fox AS, Winter JJ, Davidson RJ. The integration of negative affect, pain and cognitive control in the cingulate cortex. Nat Rev Neurosci. Nature Publishing Group; 2011;12: 154–167.

5. Ullsperger M, Danielmeier C, Jocham G. Neurophysiology of performance monitoring and adaptive behavior. Physiol Rev. Am Physiological Soc; 2014;94: 35–79.

6. Cavanagh JF, Frank MJ. Frontal theta as a mechanism for cognitive control. Trends Cogn Sci. Elsevier; 2014;18: 414–421.

7. Oades RD, Halliday GM. Ventral tegmental (A10) system: neurobiology. 1. Anatomy and connectivity. Brain Res Rev. Elsevier; 1987;12: 117–165.

8. Jay TM. Dopamine: a potential substrate for synaptic plasticity and memory mechanisms. Prog Neurobiol. Elsevier; 2003;69: 375–390.

9. Sutton RS, Barto AG. Reinforcement learning: An introduction. Cambridge, MA, USA: MIT press; 1998.

10. Schultz W, Dayan P, Montague R. A Neural Substrate of Prediction and Reward. Science (80- ). American Association for the Advancement of Science; 1997;275: 1593–1599. doi:doi: 10.1126/science.275.5306.1593

11. Shahnazian D, Holroyd C. Recurrent Neural Network Modeling of Anterior Cingulate Function. In: Pineau J, Dayan P, editors. The second Multi-disciplinary Conference on Reinforcement Learning and Decision Making.

12. Ma L, Hyman JM, Phillips AG, Seamans JK. Tracking progress toward a goal in corticostriatal ensembles. J Neurosci. Soc Neuroscience; 2014;34: 2244–2253.

13. Holroyd CB, Yeung N. Motivation of extended behaviors by anterior cingulate cortex. Trends Cogn Sci. Elsevier; 2012;16: 122–128.

14. Botvinick MM, Weinstein A. Model-based hierarchical reinforcement learning and human action control. Philos Trans R Soc B Biol Sci. The Royal Society; 2014;369: 20130480.
